# The Dominant Role of Community Influences over Individual Contraceptive Choices in Pakistan

**DOI:** 10.12688/gatesopenres.16390.1

**Published:** 2026-07-06

**Authors:** Olan Naz, Muhammad Ibrahim, Aisha Irum, Ayesha Khan, Adnan Ahmad Khan

**Affiliations:** 1 Research and Development Solutions (RADS), Islamabad, 44220, Pakistan; 2 Akhter Hameed Khan Foundation (AHKF), Islamabad, 44220, Pakistan

**Keywords:** Family planning, contraceptive use, community influence, social diffusion, women’s autonomy, LMIC, reproductive health.

## Abstract

**Introduction:**

Contraceptive use is essential for improving maternal and child health, yet family planning (FP) uptake in Pakistan remains low, with stark disparities shaped by both individual and community-level influences. While individual determinants such as education and wealth have been widely studied, the role of community norms, peer influence, and social networks in shaping contraceptive behaviors remains underexplored. Drawing on Rogers’ Diffusion of Innovation Theory this study examines how social learning and collective behaviors influence FP adoption.

**Methodology:**

This study analyzed data from the 2017–18 Pakistan Demographic and Health Survey, including 15,068 married women aged 15–49 nested within primary sampling units. Logistic regression models were estimated for any contraceptive use and modern contraceptive use. Individual-level covariates included age, education, wealth, parity, and decision-making autonomy. Community-level variables—constructed using leave-one-out means—captured shared intention to use family planning, parity norms, health-seeking behavior, wealth, age structure, and mean age at first birth. Predictive margins were used to assess non-linear relationships and potential threshold effects, with robustness assessed using a 75% subsample.

**Results:**

Community intention to use family planning was a strong and consistent predictor of contraceptive behavior. Compared to women in the lowest-intention communities, those residing in medium–high intention communities had significantly higher odds of contraceptive use (OR = 1.86; 95% CI [1.57, 2.21]) and communities with highest intention to use exhibited (OR = 1.92; 95% CI [1.61, 2.29]). At the individual level, education and wealth remained strong predictors, autonomy was modestly significant, its effect was amplified in supportive community environments where FP was normalized.

**Conclusion:**

This study underscores the transformative role of community dynamics in shaping contraceptive behaviors in Pakistan. Family planning programs may achieve greater and more durable impact by prioritizing community-wide norm formation and health system engagement rather than focusing solely on individual-level interventions.

## Introduction

Communities are powerful agents of change, shaping norms, behaviors, and decisions that form super-influences against which individuals or families decide. In family planning (FP), community dynamics create environments that can either enable or constrain contraceptive use, significantly influencing reproductive health outcomes. ^1^ In many societies, particularly in low- and middle-income countries (LMIC), individual contraceptive choices are deeply embedded within cultural and social structures. Communities influence FP behaviors through peer interactions, shared beliefs, and the diffusion of contraceptive knowledge, creating environments that either encourage or discourage the use of modern contraceptives. Higher community contraceptive prevalence rates (CPR) can reinforce contraceptive adoption by reducing stigma, normalizing FP use, and increasing awareness through social learning and peer influence. ^2^

This process of community diffusion plays a crucial role in understanding how contraceptive behaviors spread within populations. Contraceptive adoption patterns often remain socially and culturally clustered, transcending economic or political boundaries to spread through shared linguistic, ethnic, and religious affiliations. ^3^ While wealth, education, and exposure to external ideas through media or migration are associated with higher contraceptive use, evidence shows that these factors alone cannot fully explain adoption patterns. Instead, social interactions within networks and communities add another layer of influence, enabling individuals to access information, observe role models, and normalize FP behaviors. These dynamics often result in rapid behavioral shifts once critical levels of social acceptance are reached. ^4^^–^^6^

Despite the recognized importance of community-level factors, disparities in FP adoption persist globally, particularly in LMIC. In high-income countries, fertility and mortality reductions occurred in tandem, facilitated by improved healthcare systems, widespread education, and economic development. During Europe’s demographic transition (1870–1960), mortality and fertility declines were bolstered by the normalization of contraceptive use. ^7^ However, in LMIC, fertility declines have lagged behind reductions in mortality, largely due to persistent socio-economic inequalities, inadequate healthcare infrastructure, and entrenched cultural norms. ^8^ Pakistan exemplifies this disparity, with a CPR of only 34% and modern contraceptive use among married women at 25%, compared to 60% in countries like Kenya. Additionally, 8% of adolescent women aged 15–19 have already begun childbearing, highlighting systemic barriers faced by rural, less educated, and economically disadvantaged women. ^9^

The Demographic Transition Theory offers a framework for understanding fertility decline as a process influenced by both structural and socio-cultural changes. These disparities underline the need to view fertility dynamics not merely as responses to economic development but as reflections of evolving social and cultural contexts. The Diffusion of Innovations Theory (Rogers, 1962) further explains how behaviors such as contraceptive use spread within communities. According to this theory, new behaviors are adopted through interpersonal interactions and societal changes, with early adopters serving as catalysts for broader acceptance. ^10^ Coale’s (1973) framework complements this perspective by identifying three prerequisites for fertility reduction: perceived advantages of fertility control, readiness to adopt contraceptives, and access to reliable methods. These conditions are significantly shaped by community norms and social networks, which increase awareness, reduce stigma, and normalize FP practices. ^11^^,^^12^ The Health Belief Model (HBM) also provides valuable insights, emphasizing that perceived risks, benefits, and societal expectations strongly influence health behaviors, including FP adoption. ^13^^,^^14^ Moreover, social contagion theories suggest that as more women adopt modern contraceptives, the behavior becomes a community norm, reducing barriers for others to follow suit. ^15^ The “tipping point” described in social diffusion literature highlights how critical mass adoption within a community can lead to rapid behavior change among individuals. Such community-level effects often outweigh individual-level barriers such as lower autonomy or lack of education, indicating the profound role of collective behavior in FP adoption. ^16^^,^^17^

Pakistan is currently undergoing a fertility transition, where children are increasingly valued for their potential and well-being, rather than merely as economic assets. ^18^ However, how these new perceptions are disseminated through social networks and reinforced by community norms remains an open question. In this context, cultural norms and traditional joint family structures complicate the adoption of contraceptive practices. Fertility decisions are often controlled by elders, spouses, and community leaders, limiting women’s autonomy and their access to modern contraceptive methods. ^19^^,^^20^ Additionally, persistent social stigma, misinformation, and religious misconceptions reinforce community-driven barriers to FP adoption. ^21^^,^^22^ Despite substantial investments in FP programs, Pakistan’s CPR has stagnated, indicating that traditional interventions focused solely on knowledge dissemination and service provision may be insufficient. While government and donor initiatives increasingly emphasize behavior change communication (BCC) strategies, these efforts often fail to incorporate the social reinforcement mechanisms necessary to translate awareness into lasting behavior change.

Given the deep-rooted influence of community structures on FP adoption, it is imperative to examine how social networks facilitate or hinder contraceptive uptake. This study aims to explore how community’s behavior shapes individual FP behaviors in Pakistan, providing insights into the roles of social networks, cultural norms, and collective decision-making in influencing contraceptive adoption.

## Methodology

This study utilizes data from the 2017–18 Pakistan Demographic and Health Survey (PDHS), a nationally representative cross-sectional survey providing detailed information on fertility, family planning, maternal and child health, and community-level indicators. The PDHS employed a multistage stratified sampling design, where enumeration blocks, referred to as Primary Sampling Units (PSUs), were selected with probability proportional to size. PSUs represent clusters of households within a specific geographic area. Within each PSU, households were randomly sampled, and 15,068 eligible women of reproductive age (15–49 years) were interviewed.

The analysis focused on contraceptive use as the dependent variable, coded as “1” for women currently using any form of contraception and “0” for those not using contraception. Similarly, modern contraceptive use was used another dependent variable, coded as “1” for women currently using any form of modern contraception and “0” for those not using modern contraception. Independent variables were categorized into individual- and community-level factors. At the individual level, predictors included demographic characteristics such as age and the number of living children, as well as socioeconomic factors like educational attainment (no education, primary, secondary, or higher) and wealth quintiles (poorest to richest). Women’s decision-making autonomy was also included, measured through an index assessing decision-making on healthcare, purchases, and family visits, categorized into quintiles ranging from 0% to 100%.

The analysis incorporated a set of community-level variables constructed at the PSU level using a leave-one-out (LOO) approach to capture contextual influences on contraceptive behavior while minimizing mechanical correlation with individual characteristics. ^23^^,^^24^ Community health-seeking behavior (HSB) was derived from a composite index reflecting antenatal care utilization and recent health facility visits, aggregated using LOO means and similarly categorized into quartiles. Community parity norms were captured using the LOO mean number of ideal children for families within the PSU and grouped into quartiles to reflect prevailing fertility expectations. Community intention to use family planning was defined as the LOO proportion of women reporting an intention to adopt contraception in the future and categorized into quartiles to represent varying normative climates of contraceptive intent. Community economic context was measured through the LOO mean household wealth index and categorized into quartiles. In addition, community mean age at first birth was calculated from cleaned values of age at first birth using the LOO approach and retained as a continuous variable to preserve information on timing norms. Similarly, community age structure was measured as the LOO mean age of women within each PSU.

Variance inflation factors (VIFs) were examined to assess multicollinearity for both CPR and mCPR models. Mean VIF values were approximately 2.2, and all covariates were below standard cut-off values, suggesting negligible multicollinearity.

Initially, a multilevel logistic regression model was employed to account for the hierarchical data structure, with individuals nested within PSUs. However, the intra-cluster correlation coefficient (ICC) was calculated for contraceptive use, yielding a low ICC value of 8.1% [95% CI: 6.6%–10.0%]. Similarly, the ICC for modern contraceptive use was 6.8% [95% CI: 5.3%-8.7%]. This result indicates that only a small portion of the total variation is attributable to clustering at the PSU level, with most variation explained at the individual level. Additionally, the variance of the random effects at the cluster level was modest for contraceptive use (OR = 0.29; 95% CI [0.23-0.37]) and for modern contraceptive use (OR = 0.24; 95% CI [0.19-0.31]). Given the principle of parsimony and the low ICC, a standard logistic regression model was more appropriate for the analysis. The general model structure is specified as:

$$\text{Logit}(P(Y_{\mathrm{ij}}=1))=\beta_{0}+\sum \beta_{k}\;X_{\mathrm{kij}}+\sum \gamma_{l}\;Z_{\mathrm{lij}}+u_{j}\;$$ (i)

Where:

•
$Y_{\mathrm{ij}}$ = Contraceptive use for individual i in PSU j .
•
$X_{\mathrm{kij}}$ = Individual-level predictors.
•
$Z_{\mathrm{lij}}$ = Community-level predictors.
•
$u_{j}\;$ = Error term.

### Threshold modeling/Estimation

The logistic regression model calculates marginal effects or predicted probabilities to estimate the threshold at which community-level influences on FP utilization become significant. This analysis helps to identify the point at which community-level factors substantially influence the probability of FP utilization. Predicted probabilities of contraceptive use were calculated and margin plots were generated to visualize the effects of key variables, including community intent to use contraceptive, ideal number of children, wealth index, etc.

### Sensitivity analysis

Post-regression, sensitivity analysis was conducted to assess the robustness of results to different model specifications, assess the impact of excluding potential outliers, and evaluate collinearity between independent predictors, particularly focusing on the correlation of community contraceptives and the dependent variable. To evaluate this, a 75% sample sensitivity analysis was conducted. This approach involved randomly selecting 75% of the total sample and re-estimating the regression models to verify the stability and robustness of the results.

All analyses were conducted using Stata (version 17) for statistical modeling, data management, and visualization.

## Results

The dataset includes 15,068 MWRA from across Pakistan, with 40% aged 24–33, aligning with peak reproductive years. More than half (50.62%) have no formal education, while only 15% have attained higher education, reflecting barriers to knowledge and health service access. Health-seeking behavior is encouraging, with 70% demonstrating high engagement, though 6.5% report no engagement. Only 29.1% of women have full decision-making autonomy, while 39% report no autonomy. All community-level variables were categorized into quartiles to facilitate interpretation, except for community mean age at first birth, which was retained as a continuous measure. The mean age of women of reproductive age within communities was 32.3 years, while the average community mean age at first birth was 21.1 years ( Table 1).

**Table 1.** Descriptive statistics.

Variable	Percent
**MWRA’s Age in years**	
15-23	16.2
24-33	40.0
34-43	31.6
44-49	12.3
**MWRA’s Education**	
No formal education	50.6
Primary	14.0
Secondary	20.8
Higher	14.6
**Region**	
Rural	51.9
Urban	48.1
**Current Contraceptive use**	
No	68.7
Yes	31.3
**Health Seeking Index**	
0	6.5
0.5	23.2
1	70.3
**Autonomy Index**	
0%	39.2
25%	12.1
50%	9.1
75%	10.5
100%	29.1
**Wealth Index**	
Poorest	19.2
Poorer	21.5
Middle	19.7
Richer	19.1
Richest	20.6
**Community MWRA Mean Age**	32.3
**Community MWRA Mean Age at First Birth**	21.1
**Community Intent to Contraceptive use**	
Lowest Intention Norm	25.3
Low-Medium Intention Norm	25.7
Medium-High Intention Norm	24.6
Highest Intention Norm	24.5
**Community Mean Health Seeking Behavior (HSB)**	
Lowest (HSB)	25.0
Low-Medium HSB	25.2
Medium-High HSB	25.5
Highest HSB	24.3
**Community Parity Norm**	
Lowest Parity Norm	25.0
Low-Medium Parity Norm	25.1
Medium-High Parity Norm	25.0
Highest Parity Norm	25.0
**Community Mean Wealth Index**	
Poorest Communities	25.4
Lower-Middle Communities	24.7
Middle-Upper Communities	25.2
Richest Communities	24.8

Several individual-level characteristics are significantly associated with contraceptive use ( Table 2). Compared to women aged 15–24 years, young adults aged 25–34 had higher odds of contraceptive use (OR = 1.378; 95% CI [1.192, 1.593]) and modern contraceptive use (OR = 1.301; 95% CI [1.109, 1.526]), while women aged 35–44 also showed elevated odds for any contraceptive use (OR = 1.245; 95% CI [1.057, 1.467]). Autonomy, measured through a decision-making index, has a modest but consistent association with contraceptive use. Women with 50% autonomy show higher odds (OR = 1.185; 95% CI [1.021,1.375]), with similar patterns observed at 75% (OR = 1.199; 95% CI [1.041,1.380]) and 100% autonomy (OR = 1.188; 95% CI [1.066,1.324]). While these results suggest that greater decision-making power improves uptake, the gains plateau beyond moderate levels—indicating that the impact of personal agency becomes less meaningful once a threshold of autonomy is reached.

**Table 2.** Multilevel logistic regression.

Dependent variable	(I) Contraceptive use	(II) Modern contraceptive	(III) 75% sample – Contraceptive use	(IV) 75% sample – Modern Contraceptive
Mother’s Age [Base: (15-24)]				
Young Adults (25-34)	1.378*	1.301*	1.456*	1.394*
	[1.192,1.593]	[1.109,1.526]	[1.228,1.726]	[1.156,1.682]
Adults (35-44)	1.245*	1.179	1.326*	1.260*
	[1.057,1.467]	[0.986,1.410]	[1.095,1.607]	[1.022,1.552]
Older Adults (45-49)	0.633*	0.641*	0.654*	0.670*
	[0.517,0.774]	[0.515,0.798]	[0.516,0.827]	[0.519,0.864]
Mother’s Education [Base: No Formal Education]				
Primary	1.372*	1.324*	1.424*	1.356*
	[1.206,1.561]	[1.156,1.516]	[1.227,1.653]	[1.160,1.585]
Secondary	1.542*	1.468*	1.623*	1.472*
	[1.362,1.747]	[1.287,1.674]	[1.405,1.874]	[1.265,1.712]
Higher	1.773*	1.710*	1.830*	1.617*
	[1.528,2.058]	[1.461,2.002]	[1.538,2.176]	[1.347,1.942]
Wealth Quintiles [Base: Poor]				
Lower Middle	1.470*	1.391*	1.473*	1.431*
	[1.252,1.725]	[1.176,1.645]	[1.224,1.772]	[1.180,1.735]
Middle	1.853*	1.656*	1.854*	1.649*
	[1.550,2.216]	[1.372,1.999]	[1.508,2.280]	[1.328,2.048]
Upper Middle	1.941*	1.716*	1.923*	1.662*
	[1.596,2.360]	[1.397,2.108]	[1.534,2.412]	[1.311,2.107]
Rich	2.084*	1.832*	2.070*	1.863*
	[1.683,2.580]	[1.463,2.294]	[1.617,2.651]	[1.438,2.413]
Autonomy Index				
25%	1.101	1.197*	1.081	1.210*
	[0.961,1.262]	[1.037,1.382]	[0.923,1.267]	[1.024,1.429]
50%	1.185*	1.214*	1.195*	1.239*
	[1.021,1.375]	[1.038,1.421]	[1.005,1.421]	[1.033,1.486]
75%	1.199*	1.255*	1.338*	1.397*
	[1.041,1.380]	[1.083,1.455]	[1.138,1.573]	[1.181,1.653]
100%	1.188*	1.222*	1.199*	1.240*
	[1.066,1.324]	[1.091,1.370]	[1.058,1.358]	[1.088,1.414]
Number of living children				
	1.505*	1.425*	1.511*	1.419*
	[1.468,1.544]	[1.388,1.462]	[1.467,1.555]	[1.378,1.462]
Region [Base: Urban]				
Rural	1.097	1.137	1.099	1.117
	[0.939,1.282]	[0.976,1.324]	[0.928,1.302]	[0.949,1.315]
Community Contraceptive Intention Norm [Base: Lowest]				
Low-medium intention norm	1.446*	1.509*	1.386*	1.445*
	[1.229,1.702]	[1.281,1.778]	[1.158,1.659]	[1.208,1.729]
Medium-high intention norm	1.861*	1.718*	1.816*	1.708*
	[1.571,2.205]	[1.450,2.036]	[1.506,2.190]	[1.419,2.054]
Highest intention norm	1.920*	1.763*	1.828*	1.648*
	[1.613,2.287]	[1.481,2.098]	[1.508,2.215]	[1.364,1.990]
Community Parity Norm (Base: Lowest)				
Low-medium parity norm	0.879	0.888	0.885	0.885
	[0.755,1.023]	[0.762,1.035]	[0.747,1.049]	[0.748,1.048]
Medium-high parity norm	0.819*	0.850	0.779*	0.822
	[0.682,0.983]	[0.708,1.021]	[0.636,0.955]	[0.672,1.004]
Highest community parity norm	0.758*	0.715*	0.775*	0.756*
	[0.611,0.940]	[0.577,0.887]	[0.611,0.983]	[0.598,0.955]
Community Wealth (Base: Poorest)				
Lower-middle wealth	1.341*	1.122	1.322*	1.097
	[1.099,1.636]	[0.920,1.368]	[1.060,1.647]	[0.883,1.362]
Upper-middle wealth	1.563*	1.192	1.598*	1.227
	[1.233,1.981]	[0.941,1.511]	[1.229,2.078]	[0.947,1.590]
Richest communities (highest wealth)	2.257*	1.502*	2.235*	1.485*
	[1.709,2.980]	[1.137,1.982]	[1.641,3.043]	[1.095,2.014]
Community Health-Seeking Behavior (Base: Lowest)				
Low-medium community health-seeking behavior	1.329*	1.404*	1.339*	1.434*
	[1.117,1.582]	[1.179,1.672]	[1.103,1.625]	[1.183,1.737]
Medium-high community health-seeking behavior	1.310*	1.317*	1.310*	1.319*
	[1.087,1.578]	[1.094,1.585]	[1.066,1.609]	[1.077,1.615]
Highest community health-seeking behavior	1.261*	1.276*	1.315*	1.427*
	[1.035,1.537]	[1.048,1.553]	[1.057,1.635]	[1.153,1.767]
Community mean age at first birth (years)	0.940*	0.934*	0.947	0.945
	[0.888,0.995]	[0.883,0.988]	[0.890,1.008]	[0.890,1.005]
Community mean age (years)	1.066*	1.071*	1.055*	1.052*
	[1.025,1.108]	[1.031,1.114]	[1.010,1.101]	[1.009,1.097]
Random Effects Cluster-level variance	1.338*	1.273*	1.365*	1.261*
	[1.252,1.430]	[1.195,1.355]	[1.260,1.478]	[1.173,1.357]
Observations	14379	14379	10863	10863

Community-level factors also show significant associations with contraceptive behavior. Women living in communities with the highest intention to use family planning had markedly higher odds of contraceptive use (OR = 1.920; 95% CI [1.613, 2.287]) and modern method use (OR = 1.763; 95% CI [1.481, 2.098]) compared to those in the lowest intention communities. In contrast, residing in communities with the highest parity norms was associated with lower odds of contraceptive use (OR = 0.758; 95% CI [0.611, 0.940]) and modern contraceptive use (OR = 0.715; 95% CI [0.577, 0.887]). Community wealth also mattered: women in the richest communities had significantly higher odds of contraceptive use (OR = 2.257; 95% CI [1.709, 2.980]) and modern contraceptive use (OR = 1.502; 95% CI [1.137, 1.982]). Higher levels of community health-seeking behavior were positively associated with use, particularly in the highest quartile (OR = 1.261; 95% CI [1.035, 1.537]). Community mean age was positively associated with contraceptive use (OR = 1.066; 95% CI [1.025, 1.108]), whereas higher community mean age at first birth was modestly negatively associated (OR = 0.940; 95% CI [0.888, 0.995]).

**Figure 1.**
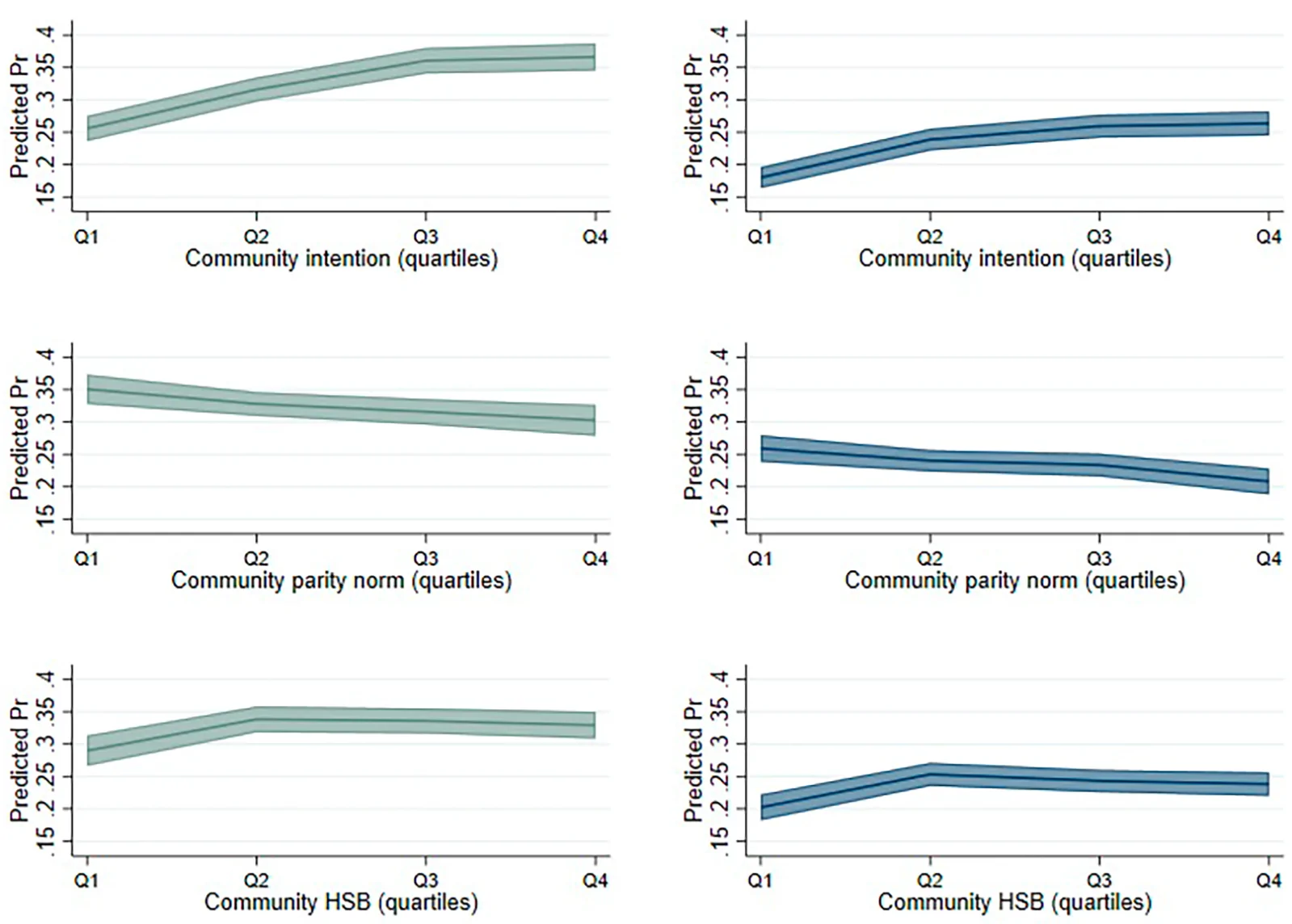
Impact of community intention to use contraceptive, mean ideal number of children (parity norm), and health-seeking behavior on predicted mean of contraceptive utilization.

These patterns are further supported by predictive margins plots ( Figure 1). As community intention to use family planning increases from the lowest to the highest quartile, the predicted probability of individual contraceptive use rises steadily. This gradient is stronger for overall contraceptive use than for modern methods, indicating that intention norms translate more readily into any method use, while adoption of modern methods remains comparatively constrained. In contrast, higher community parity norms are associated with a consistent decline in contraceptive use: women residing in communities with the highest average number of children exhibit the lowest predicted probabilities of both CPR and modern CPR, highlighting the persistence of high-fertility norms that discourage contraceptive uptake. Community health-seeking behavior shows a positive association with contraceptive use, particularly between the lowest and middle quartiles, after which gains plateau—suggesting diminishing marginal returns once a basic level of healthcare engagement is established. Across all community factors, the magnitude of effects is systematically larger for overall CPR than for modern CPR, underscoring that while community norms and behaviors strongly shape contraceptive use in general, additional barriers may limit the translation of favorable community environments into modern method adoption.

**Figure 2.**
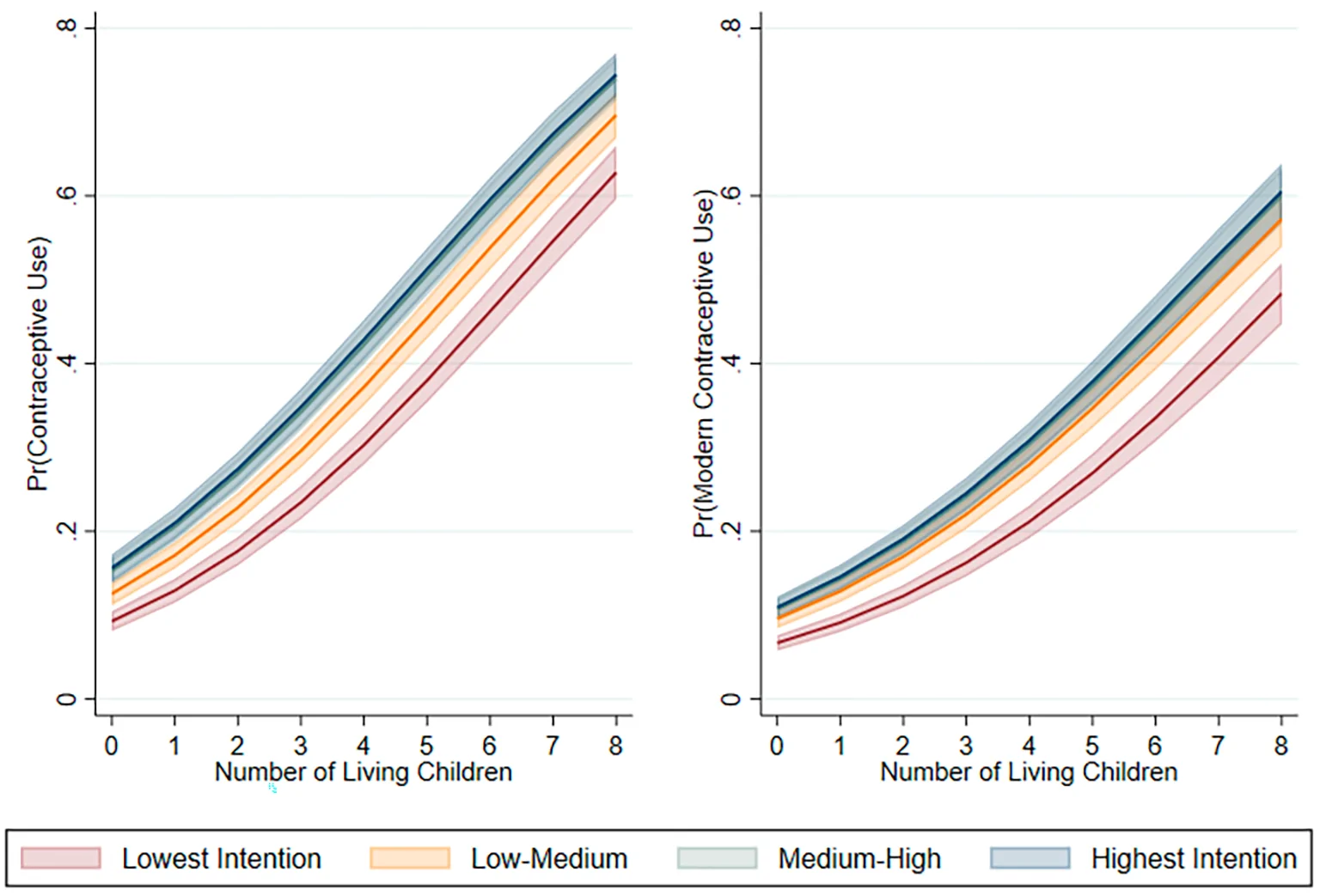
Number of living children and community intention to use contraceptive with predicted probability of contraceptive use.

The predictive margins plot illustrates the relationship between the number of living children and individual contraceptive use (CPR), stratified by different levels of community intention to use family planning ( Figure 2). The predictive margins plot illustrates the relationship between the number of living children and individual contraceptive use, stratified by levels of community intention to use family planning. Across all parity levels, women residing in communities with higher intention to use consistently exhibit higher predicted probabilities of both overall contraceptive use and modern contraceptive use. The probability of contraceptive use increases sharply with each additional child; however, the gradient is steeper in communities characterized by stronger intention norms, indicating an amplifying interaction between individual fertility experience and community-level demand for family planning. Notably, the predicted probabilities for the “Medium–High” and “Highest” intention quartiles show substantial overlap across much of the parity distribution, suggesting diminishing marginal gains in contraceptive uptake once community intention surpasses a high threshold. While the overall pattern is similar for modern methods, predicted probabilities remain uniformly lower than for any-method use, indicating that parity-driven demand translates more strongly into overall contraceptive adoption than into modern method uptake.

**Figure 3.**
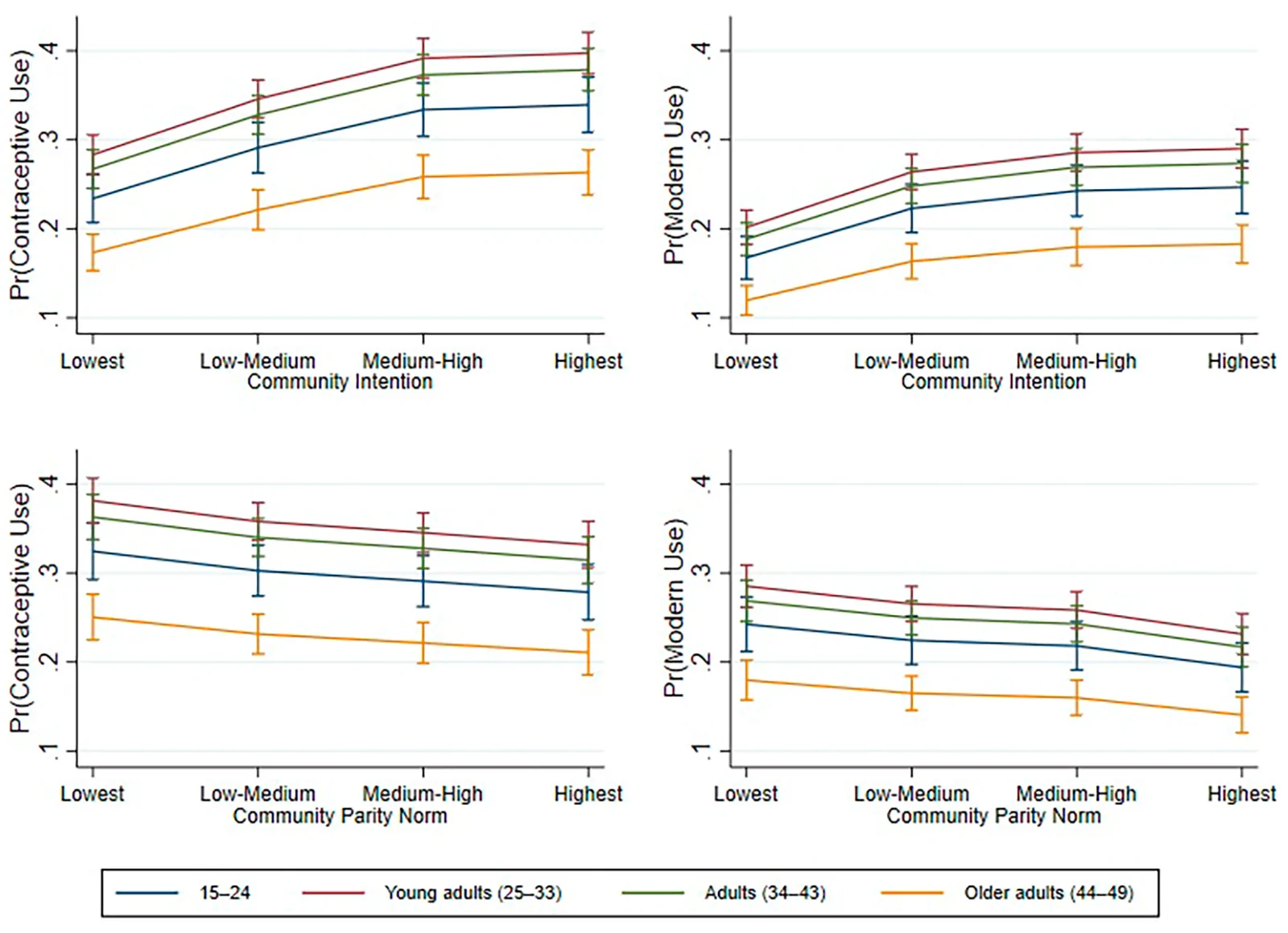
MWRA age and community intention to use contraceptive with predicted probability of individual contraceptive use.

Across all age groups, community intention to use family planning is positively associated with higher predicted probabilities of both overall contraceptive use and modern contraceptive use ( Figure 3). The upward gradient is observed consistently from the “Lowest” to the “Highest” intention quartiles, indicating that stronger community demand norms amplify individual uptake across the life course. The association is most pronounced among young adults (25–34) and adults (35–44), who exhibit the highest predicted probabilities at elevated levels of community intention, suggesting greater responsiveness to community-level normative environments during peak reproductive ages. In contrast, older women (44–49) display lower overall probabilities and a flatter gradient, indicating weaker sensitivity to community intention norms. A similar pattern is observed for modern contraceptive use, although predicted probabilities are uniformly lower and the slopes are more gradual. Notably, the confidence intervals for the “Medium–High” and “Highest” intention quartiles overlap across most age groups, suggesting that gains in contraceptive use plateau once community intention reaches high levels.

**Figure 4.**
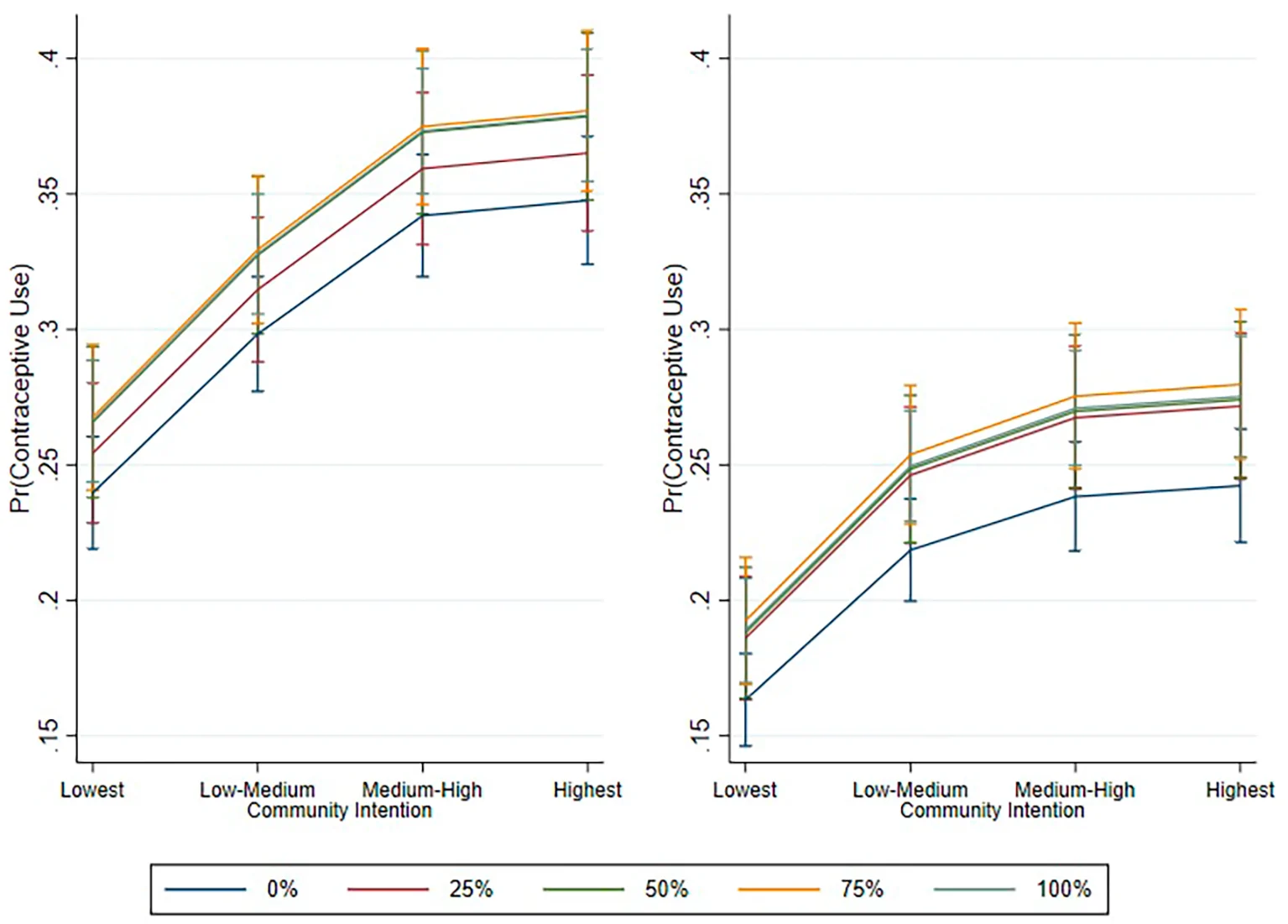
Women’s autonomy and community intention to use contraceptive with predicted probability of individual contraceptive use.

The predictive margins illustrate how community intention to use family planning shapes individual contraceptive behavior across varying levels of women’s autonomy. For both overall contraceptive use and modern contraceptive use, higher community intention is consistently associated with higher predicted probabilities of adoption at every level of autonomy. Women residing in communities classified as “Medium–High” and “Highest” intention exhibit the greatest likelihood of contraceptive use, even among those with low autonomy, underscoring the dominant role of community normative environments. While increases in autonomy are associated with modest gains in contraceptive use, the slope flattens at higher autonomy levels, indicating diminishing marginal returns once a threshold of decision-making power is reached. This pattern is evident for both CPR and modern CPR, suggesting that strong community intention can partially compensate for limited individual autonomy in shaping contraceptive behavior ( Figure 4).

## Discussion

Community-level norms emerge as the dominant predictors of contraceptive behavior. Women living in communities with higher intention to use family planning had markedly greater odds of contraceptive uptake, rising from the low-medium (OR = 1.45; 95% CI: 1.23–1.70) to medium-high (OR = 1.86; 95% CI: 1.57–2.21) and highest intention quartiles (OR = 1.92; 95% CI: 1.61–2.29), with a similar though attenuated pattern for modern methods. Marginal effects indicate steep gains across intention quartiles, with convergence between the medium-high and highest categories, suggesting diminishing marginal returns once community acceptance is widespread. In contrast, stronger community parity norms were inversely associated with contraceptive use, with women in the highest parity-norm communities experiencing 24% lower odds of use (OR = 0.76; 95% CI: 0.61–0.94). Higher community health-seeking behavior independently increased contraceptive uptake (ORs ≈ 1.26–1.33), while higher community mean age at first birth was associated with reduced use (OR ≈ 0.94 per year). Interaction margins show that these community effects persist across age, parity, and autonomy levels, indicating that contextual norms shape individual behavior even among women with greater autonomy or higher parity. Results remain stable in 75% subsample analyses, supporting the robustness of community-level influences and suggesting the presence of a social tipping point for family planning adoption.

These patterns are consistent with Coale’s Preconditions for Fertility Decline, underscoring that individual agency operates most effectively when embedded within enabling social environments. While education, wealth, and autonomy remain important individual determinants, the margin plots demonstrate that community-level intention to use family planning exerts a dominant and monotonic influence on contraceptive behavior, for both overall and modern methods. In contrast, community parity norms show a dampening effect, and community health-seeking behavior exhibits a positive but more modest association, suggesting that not all collective characteristics exert equal normative force. Crucially, the persistence of higher predicted contraceptive use at elevated levels of community intention—even among women with low autonomy—indicates that collective norms can partially substitute for constrained individual agency. This lends strong support to diffusion and social learning theories, whereby visible adoption within communities lowers social costs, reduces uncertainty, and normalizes contraceptive behavior. ^25^ Similar mechanisms have been documented in other LMIC settings, including Kenya, where peer effects and normative endorsement have been shown to accelerate family planning uptake, reinforcing the centrality of community context in shaping reproductive behavior. ^14, 26^

Community health-seeking behavior exhibits a notably faster tipping point than other community norms, with substantial gains in contraceptive use occurring between the lowest and low–medium HSB quartiles, after which the marginal increases flatten. The margin plots indicate that once routine engagement with health services becomes established within a community, contraceptive adoption rises quickly and stabilizes, suggesting that even modest improvements in collective health-seeking can unlock large behavioral returns. This underscores the importance of embedding family planning within existing health system touchpoints—such as antenatal care, immunization visits, and primary care consultations—particularly in norm-conservative settings where direct FP messaging may face resistance. For implementers, these findings imply that strengthening general health system engagement may be one of the most efficient pathways to catalyzing and sustaining contraceptive uptake at scale. ^27^

The positive association between community mean age and contraceptive use indicates that older communities in Pakistan may reflect cumulative exposure to family planning programs and greater normalization of contraceptive behavior rather than resistance to adoption. Given that contraceptive use in Pakistan is concentrated among women over age 30 and often initiated after achieving higher parity, communities with higher mean age likely embody shared experiential knowledge, peer reassurance, and reduced social stigma around family planning. ^2^ Building on this, the following findings highlight how shifts in community intention—once reaching a critical threshold—can further amplify contraceptive uptake even among women with limited individual autonomy, underscoring the central role of community dynamics in sustaining behavior change.

While women’s autonomy remains an important enabler of contraceptive use, its effects are strongly shaped by the surrounding community context. The findings indicate that autonomy generates the greatest gains in settings where supportive family planning norms are already present, but yields only modest returns in communities with weak intention to use contraception. This underscores the limits of individual empowerment strategies in socially unsupportive environments and highlights that decision-making power does not operate in isolation. Consistent with social learning and diffusion theories, women—particularly those facing uncertainty or social constraints—appear to rely on visible community behaviors and normative signals when making reproductive choices. From a policy perspective, this suggests that investments in women’s autonomy must be paired with deliberate efforts to shift community norms; otherwise, empowerment gains risk being muted. Interventions that simultaneously strengthen women’s agency and cultivate collective endorsement of family planning are therefore more likely to produce durable increases in contraceptive uptake, as supported by global evidence from LMIC. ^28– 30^

The margin plots further point to a potential tipping point in community intention to use family planning, beyond which individual contraceptive adoption rises rapidly and then begins to level off. Once communities reach the medium–high intention range, additional increases in individual resources or autonomy deliver diminishing marginal returns, suggesting that normative acceptance becomes self-reinforcing. For policymakers, this underscores the strategic importance of sustaining community-level interventions until such thresholds are achieved, after which contraceptive use may diffuse organically and persist with reduced programmatic intensity.

### Limitations and future research

While this study provides valuable insights into FP dynamics in Pakistan, certain limitations must be acknowledged. The reliance on cross-sectional data limits causal inferences, and the influence of unmeasured variables, such as cultural attitudes or provider biases, cannot be ruled out. Future research should explore longitudinal data to capture temporal changes and employ mixed-method approaches to understand the nuanced interplay between individual, community, and systemic factors.

## Conclusions and policy implications

The findings highlight the power of community-driven approaches in increasing contraceptive use and call for a shift toward norm-based interventions that shift FP use at the community level. A common limitation of many programs, including those for FP, is that despite considerable work and resources, their impact is not visible in national surveys. In part, this reflects the limited coverage of these programs. ^31^ A possible solution is to conceptualize interventions at the level of communities that are big enough to show up on national surveys. If programs achieve coverage at this level and, as we show in this paper, community norms drive individual behaviors, then this would suggest sustained programming in a locality until rising CPR may become a self-sustaining reality. This would be consistent with the experience of sustained programs such as Matlab, Bangladesh, which eventually transitioned from a project to a regular government program because behavior change had become so engrained across multiple health domains till no special interventions were needed. ^32^^,^^33^

The results also point to the possibility of a tipping point in community acceptance of family planning, beyond which contraceptive use diffuses and stabilizes with reduced external input, consistent with diffusion of innovation theory. ^34^ While the precise thresholds at which such tipping points occur remain an empirical question, recognizing their potential has important implications for program design. Rather than dispersing resources thinly, policymakers may achieve greater long-term impact by concentrating efforts in selected communities until normative change is firmly established.

The greater responsiveness of younger women to community influences highlights that parity norms are more effectively shifted through collective rather than individual-level interventions. ^35^ In Pakistan, where contraceptive adoption is often delayed until higher parity is reached, communities that visibly endorse smaller family sizes can reset reproductive expectations earlier in the life course. Such norms are socially reinforced through peer interaction and shared experience, making community saturation and strong health-seeking behavior critical for sustained change. This suggests that policies aiming to alter parity norms should prioritize community-wide engagement over isolated interventions to achieve durable impacts on fertility behaviour.

## Data Availability

The Pakistan Demographic and Health Survey (PDHS) 2017–2018 dataset was previously accessible through the following link: https://dhsprogram.com/data/dataset/Pakistan_Special_2019.cfm?flag=0. However, it is no longer available for direct download from the website. Interested individuals or institutions may request access to the dataset by contacting info@resdev.org.
